# Exploring human splenic red pulp vasculature in virtual reality: details of sheathed capillaries and the open capillary network

**DOI:** 10.1007/s00418-020-01924-3

**Published:** 2020-10-19

**Authors:** Birte S. Steiniger, Henriette Pfeffer, Michael Guthe, Oleg Lobachev

**Affiliations:** 1 Institute of Anatomy and Cell Biology, University of Marburg, Robert-Koch-Str.8, 35037 Marburg, Germany; 2 Visual Computing, Institute of Computer Science, University of Bayreuth, 95440 Bayreuth, Germany; 3 Institute of Functional and Applied Anatomy, Hannover Medical School, 30625 Hannover, Germany; 4 Present Address: Leibniz-Fachhochschule School of Business, 30539 Hannover, Germany

**Keywords:** Human spleen, Capillary sheaths, 3D reconstruction, Virtual reality

## Abstract

We reconstructed serial sections of a representative adult human spleen to clarify the unknown arrangement of the splenic microvasculature, such as terminal arterioles, sheathed capillaries, the red pulp capillary network and venules. The resulting 3D model was evaluated in virtual reality (VR). Capillary sheaths often occurred after the second or third branching of a terminal arteriole and covered its capillary side or end branches. The sheaths started directly after the final smooth muscle cells of the arteriole and consisted of cuboidal CD271^++^ stromal sheath cells surrounded and infiltrated by B lymphocytes and macrophages. Some sheaths covered up to four sequential capillary bifurcations thus forming bizarre elongated structures. Each sheath had a unique form. Apart from symmetric dichotomous branchings inside the sheath, sheathed capillaries also gave off side branches, which crossed the sheath and freely ended at its surface. These side branches are likely to distribute materials from the incoming blood to sheath-associated B lymphocytes and macrophages and thus represent the first location for recognition of blood-borne antigens in the spleen. A few non-sheathed bypasses from terminal arterioles to the red pulp capillary network also exist. Red pulp venules are primarily supplied by sinuses, but they also exhibit a few connections to the capillary network. Thus, the human splenic red pulp harbors a primarily open microcirculation with a very minor closed part.

## Introduction

The red pulp of the human spleen exhibits a unique microvasculature (Steiniger and Barth 2000; Steiniger 2015). Its arterial side is composed of terminal arterioles, followed by sheathed capillaries feeding a capillary network with many open ends. The blood then passes a reticular connective tissue without endothelia and, finally, enters venous sinuses which join and form venules. The blood-containing spaces in the reticular connective tissue of splenic cords represent the open component of the splenic circulatory system. These spaces are lined by fibroblasts and macrophages. They lead the blood back into the circulation via slits between the sinus endothelial cells (Drenckhahn and Wagner 1986). Up to now, direct connections between capillaries and sinuses have not been convincingly demonstrated.

This special construction makes the splenic red pulp cords the most effective phagocytic compartment of the human body. In addition, it provides a storage compartment for platelets (Kotzé et al. 1986). It is an unsolved question how blood coagulation is prevented in the absence of endothelial cells.

Apart from the open connective tissue spaces, sheathed capillaries and sinuses are spleen-specific features which need further exploration. In previous publications (Steiniger et al. 2014, 2018), we analyzed the cell phenotype and subsequently the location and form of capillary sheaths in three dimensions, applying a limited number of serial sections. After having discovered that strong expression of CD271 could be used to identify stromal capillary sheath cells, we demonstrated sheaths as rather long structures which started in a direct post-arteriolar position and covered up to three branchings of the respective capillary. This analysis was alleviated by the fact that the endothelial antigen CD34 is primarily expressed by arterial/arteriolar, capillary and vein/venular endothelia, but is almost absent in sinus endothelia (Steiniger et al. 2007). In addition, we showed that the sheaths were surrounded by naive B lymphocytes and that the stromal sheath cells produce the B cell attracting chemokine CXCL13 (Steiniger et al. 2014).

We now reconstruct a larger series of sections and additionally visualize smooth muscle alpha-actin and B lymphocytes by separate colors. We analyze regions of interest (ROIs) in the 3D model by virtual reality (VR) and isolate single capillary sheaths and sheath systems to demonstrate their variability in shape and length. Our results indicate that only few branches of terminal arterioles connect to the red pulp capillary network without a capillary sheath and that only a limited number of capillaries finally join red pulp venules. Taken together, it is likely that most of the blood which passes the splenic red pulp runs through sheathed capillaries and through the open circulatory system of the splenic cords. This does not exclude that "emergency exits" to venules exist.

## Materials and methods

### Specimen and sections

A specimen of a 22-year-old male accident victim obtained in the year 2000 was fixed in 3.7% formaldehyde in tap water for 24 h at 4 °C, embedded in paraffin and used for cutting 150 serial sections in 2018. The acquisition was carried out in accordance with the ethical regulations at the time the sample was obtained. In 2000, an ethics vote was not obligatory for work with human materials at the medical faculty of Marburg University. This practice was retrospectively approved by the ethics committee.

The serial sections were cut with an N35 blade (Feather Safety Razor Co. Ltd., Osaka, Japan) in a Leica RM2255 microtome with a blade inclination angle of 2.5° using silanized slides. Two sections were lost. The average section thickness was 7 µm. High-temperature antigen retrieval was mandatory for immunostaining, but it caused structures containing dense connective tissue, such as splenic trabeculae, to contract and to partially detach from the slide. This led to small uneven spots in the section surface, which provoked focusing problems during automatic scanning. In consequence, only about 80–90 sections per ROI were appropriate for three-dimensional reconstruction without major digital repair measures. These sections were used in the present publication. In addition, for ROI 4, the data of an entire series of 150 sections were digitally amended (Lobachev 2020) prior to reconstruction, but only used for comparison. In detail, the following numbers of serial sections were evaluated: ROI1: 83, ROI2: 83, ROI3: 93, ROI4: 84.

### Triple staining procedure

The sections were first double stained for smooth muscle cells and certain fibroblasts using anti-smooth muscle alpha-actin (SMA, brown) in combination with endothelial cells (CD34, blue). Every other section was subsequently incubated to additionally demonstrate either resident sheath cells (CD271, red) or B lymphocytes (CD20, red).

In detail, polyclonal antibodies against smooth muscle alpha-actin (SMA, Sino Biological via Biozol, Eching, No. 100125-T40) and mAb QBend 10 against CD34 (Dianova, Hamburg, Germany, No. DLN-09135) were mixed at final dilutions of 1:14.000 and 1:5.000, respectively, using PBS/BSA/NaN_3_ containing 0.003 mg/ml avidin. The sections were autoclaved, treated with glucose oxidase (Steiniger et al. 2018) and incubated with the antibody solutions overnight at 4 °C. On the following day, the sections were warmed to room temperature, washed with PBS and covered first with biotinylated anti-rabbit IgG (Vector Labs, Burlingame CA, No. BA-1000, via Linaris, Wertheim, Germany) containing 0.02 mg/ml biotin and then, after washing, with preformed avidin-biotinylated peroxidase complexes (ABC, Vector Labs No. PK-6100, via Linaris, Wertheim, Germany) according to the instructions of the manufacturer. The presence of peroxidase was revealed in brown by a benzidine reaction to show the distribution of SMA. Subsequently, bound QBend 10 was detected in blue by BrightVision anti-mouse IgG (ImmunoLogic, Amsterdam, Netherlands via VWR, Darmstadt, Germany, No. VWRKDPVM55AP) and Enzo HighDef Blue for AP (Enzo Life Sciences, Lörrach, Germany, No. ADI-950-150) as chromogen. In the third step, either anti-CD271 mAb EP1039Y (GeneTex via Biozol, Eching, Germany, No. GTX61425) was applied at 1:600 overnight and revealed in red by BrightVision anti-rabbit IgG (ImmunoLogic, Amsterdam, Netherlands via VWR, Darmstadt, Germany, No. VWRKDPVR55AP) and Enzo HighDef Red (Enzo, Lörrach, Germany, No. ADI-950-140-0030) or anti-CD20 mAb L26 (DAKO, Hamburg, Germany, No. M0755) was applied at 1:500 and incubated for 1 h at room temperature. CD20 was demonstrated by BrightVision anti-mouse IgG and Enzo HighDef Red. All slides were coverslipped in Mowiol (Sigma-Aldrich, No. 324590).

All antibodies were carefully titrated for use in triple-staining procedures. Omission of each of the antibodies had previously shown that non-specific background staining by the detection systems did not occur.

### Visualization procedures—registration

The sections were scanned in 2018 using a Leica SCN 400 microscope with a 20x lens at 0.5 µm/pixel. The acquisition area was approx. 8 × 11 mm. ROIs 1, 2, and 4 were selected to include as much red pulp as possible while minimizing the presence of follicles and large blood vessels. ROI 3 was selected from a region dominated by white pulp to escape bias. All regions were normalized (Khan et al. 2014; Reinhard et al. 2001) to a single standard image. After the registration was completed, 2.5 k × 2.5 k pixels were selected in the center of the regions and used in further processing. Not all images in the ROI sequences were in focus due to partial detachment of red pulp trabeculae during high-temperature antigen retrieval. We thus had to crop the series of automatically scanned sections in the individual ROIs by about 44% to avoid excessive digital repair measures.

We applied our custom registration software (Lobachev et al. 2017b). It is a sparse global registration, meaning that it is based on feature detection in input images. The software optimizes the positions of all images as a stack instead of image pairs. Color deconvolution (Ruifrok and Johnston 2001; Onder et al. 2014) was used to extract the blue label showing CD34. This blue channel was blurred for the registration by a Gaussian blur with sigma 6 in ImageMagick (Still 2006). The resulting images were used to establish the transformations for the registration. The established transformations were then applied to the actual non-blurred images of the sections.

### Visualization procedures—3D reconstruction

Next, the actual processing for the 3D reconstruction was performed. We subtracted the background and computed a color deconvolution for brown (SMA), blue (CD34), and red (alternating, CD271 and CD20) with Fiji (Schindelin et al. 2012). All channels were converted to 8-bit intensity images. The blue staining from color deconvolution was converted to the RGB color space, negated, and only the values from the green channel were used further. Analogously, the brown staining was processed as negated blue from RGB. For the red staining, we utilized magenta from the CMYK color space. The conversions ensured the correct intensity-based representation of the data by stretching the intensity interval. This allowed for easier *iso*-value selection.

The separated channels for SMA, CD34, and CD271 (sheaths) were subjected to our custom interpolation procedure (Lobachev et al. 2017a). Basically, we used optical flow (Farnebäck 2003) to increase the resolution in the z axis (and thus to reduce the anisotropy) from 0.5 × 0.5 × 7 µm/voxel to 0.5 × 0.5 × 1 µm/voxel for SMA and CD34 and from 0.5 × 0.5 × 14 µm/voxel to 0.5 × 0.5 × 2 µm/voxel for sheaths. Thus, in the x/y plane, the initial resolution of the scans was maintained in the models. The resolution in z differs according to the antibody used (all sections were stained for CD34 and SMA, every other section was additionally stained for CD20). Thus, the resolution is 0.5 × 0.5 × 1 µm/voxel for CD34 and SMA, 0.5 × 0.5 × 2 µm/voxel for CD271 and 0.5 × 0.5 × 14 µm/voxel for CD20 (no interpolation in z).

As B lymphocytes are single round cells, no shape-connecting interpolation for this channel was needed. We resized the stack in z-dimension using Fiji and nearest neighbor interpolation. After interpolation, all volumes were cropped at the center to 2 k × 2 k pixels. A single volume for a single channel with 83 sections occupied about 2.3 GB.

The volume data of the CD34 channel were subjected to a grayscale closing filter with uniform radius 7 and to a Gaussian blur with sigma value of 1. For SMA, we applied a grayscale dilation filter with radius 7-7-4, a closing filter with radius 8-8-4, and Gaussian blur with sigma 2. The CD271 channel was dilated with radius 8-8-2, followed by a closing filter with uniform radius 5, and Gaussian blur with sigma 1. We did not further process the CD20 channel. The processing was carried out in 3D Slicer (Pieper et al. 2004; Kikinis et al. 2014). We mainly used ParaView (Ahrens et al. 2005; Ayachit 2015) for the 3D reconstruction from final volumes. We applied the iso-value 100 for CD34 and CD20, and the iso-value 160 for SMA and CD271.

### Visualization procedures—post-processing

The resulting meshes from the 3D reconstructions were extremely large (dozens of gigabytes), and hence impractical to use directly. Additionally, the marching cubes algorithm (Lorensen and Cline 1987) would leave certain surfaces open, e.g., structures at ROI boundaries. To cope with both these problems, we used PolyMender (Ju 2004) on the reconstructed meshes. We used octree depth 9 for the CD34 channel (to preserve even very thin capillaries), depth 7 for SMA (we were only interested in large structures), and depth 8 for sheaths and B cells. Prior investigation of Hausdorff distances (Alt and Guibas 1999) on meshes before and after PolyMender-induced repair had shown that such conversions did not result in any relevant shape differences (Steiniger et al. 2016). Then, further mesh processing was performed in Meshlab (Cignoni et al. 2008). The correspondence of volume data and meshes was established by quality controlling the reconstructions by means of the registered original sections.

We applied the Taubin smoothing algorithm (Taubin 1995) in standard ten iterations to all meshes except the CD20 mesh for B cells. Small non-connected components were removed at 2% of the main diagonal size (about 76 µm) for CD34 meshes, at 5% for CD271 meshes and at 10% for SMA meshes. Such small, non-connected components are a typical 3D reconstruction noise that we speculate to originate from non-stained tissue contrasts in the acquired sections. Removal was necessary, because we were only interested in large structures in the CD34 and SMA meshes. In CD20 meshes, we did not remove small components, except in Fig. 6. Finally, we applied the quadric decimation in Meshlab, yielding 10% of the mesh size for the CD34 channel and 50% for the CD271 and CD20 channels.

We pruned B lymphocytes (Fig. 6, Suppl. File 8, Suppl. Video 4) by Hausdorff distances, manual selection and removal of components less than 10 µm in diameter. Supplementary file 9 demonstrates the processed and unprocessed data.

### Visualization procedures—virtual reality

The decimated meshes were small enough to stably produce 90 fps frame rates using a Nvidia GTX 1070 graphics card and a HTC Vive virtual reality headset. Non-decimated meshes decreased the frame rate to 20 fps, which is quite straining for the user in virtual reality (VR).

We found that the SMA reconstruction, showing smooth muscle cells in larger blood vessels (and hence allowing to clearly distinguish them from capillaries) induced huge visual clutter, even with low octree levels of PolyMender, high sigma values for blurring, etc. Therefore, we decided to visualize SMA staining as a color in the CD34 blood vessel channel. Specifically, using the Hausdorff distance (Alt and Guibas 1999) between the CD34 and SMA channels, we highlighted all vertices of the CD34 mesh in white color that lay closer than 8.75 µm to the SMA mesh. All other parts of the CD34 mesh were painted blue.

Evaluation of the models was performed in VR, combining the SMA-highlighted CD34 mesh (white and blue) with the CD271 capillary sheaths mesh (green) and the CD20 B cell mesh (red). The CD34^+^ capillaries are very densely distributed, so that a 3D model of about 80 serial sections severely occludes itself. Occlusion prevents an easy and fast interpretation of details in interactive 3D desktop applications. A slice-based approach in, e.g., 3D Slicer, would allow for quality control, but not for the entire investigation performed. The decisive advantage of VR is that the observer is totally immersed in the model and that the model is not moved. This means that the brain's innate systems of spatial orientation and spatial memory can be fully exploited to alleviate recognition and annotation of structures. Thus, analyzing and highlighting structures in VR are much quicker than on a screen and less tiring. In the immersed state, the observer can continue working for hours, which is totally impossible, if a screen is used. Human innate orientation systems do not reach their full potential, if the observer stays remote from the model and needs to move it.

The high degree of structural occlusion necessitated an adjustment of our VR viewer (Berthold 2017; Lobachev 2018). In a classic computer graphics approach, the view frustum consists of six planes, also called “clipping planes”. We allowed for the user-based adjustment of front and rear planes of the view frustum. This leads to the consequence that parts of the reconstruction vanish, when the user moves. Front plane clipping is perceived as a space which is dynamically vacated in the reconstruction directly in front of the user´s eyes. The front plane clipping adjustment thus allows inspecting all inner details of the reconstruction without any effort (Fig. 8, Suppl. Video 3).

In our VR application, the users can place spherical annotations into the reconstructions by means of the Vive controller to support communication. With a similar setup involving geodesic distance computation, the users are able to colorize parts of the reconstructed meshes to highlight particular surfaces, a process called "mesh painting". Thus, it is possible to trace blood vessels from large arteries via arterioles to capillaries and to their open ends.

### Visualization procedures—quality control and labeling in VR

Our VR visualization tool allows inspecting the original (registered) sections blended into the mesh-based 3D reconstruction. This means that experts do not need to base their decisions on the model, but that they can simultaneously consult the original sections. This method also allows for the inspection of objects omitted from the reconstruction. For example, the SMA-based highlights on CD34^+^ blood vessels were used as an initial automatically generated guide for diagnosing arterioles in the 3D models.

Visualization of our models as 2D images represents a special challenge. To cope with this problem, we used mesh painting to manually label terminal arterioles or groups of arterioles as well as the capillaries inside sheaths to separate them from the surroundings. In addition, we highlighted open side branches of the central capillary in a sheath. We opted for manual labeling as automatic connection analysis would have also shown the entire capillary network fed by sheathed capillaries. Thus, only CD34^+^ endothelia, SMA^+^ areas, capillary sheaths and B lymphocytes were detected automatically.

Our interactive visualizations are provided at https://zenodo.org/record/4059595 of the Zenodo repository. Interactive visualizations are superior to videos. We suggest using these, provided the technical possibility (detailed on site) is given. They should be viewed with a VR headset for unequivocal diagnosis, but an interactive visualization is also possible without VR.

## Results

### Antigens and their distribution

**Fig. 1:**
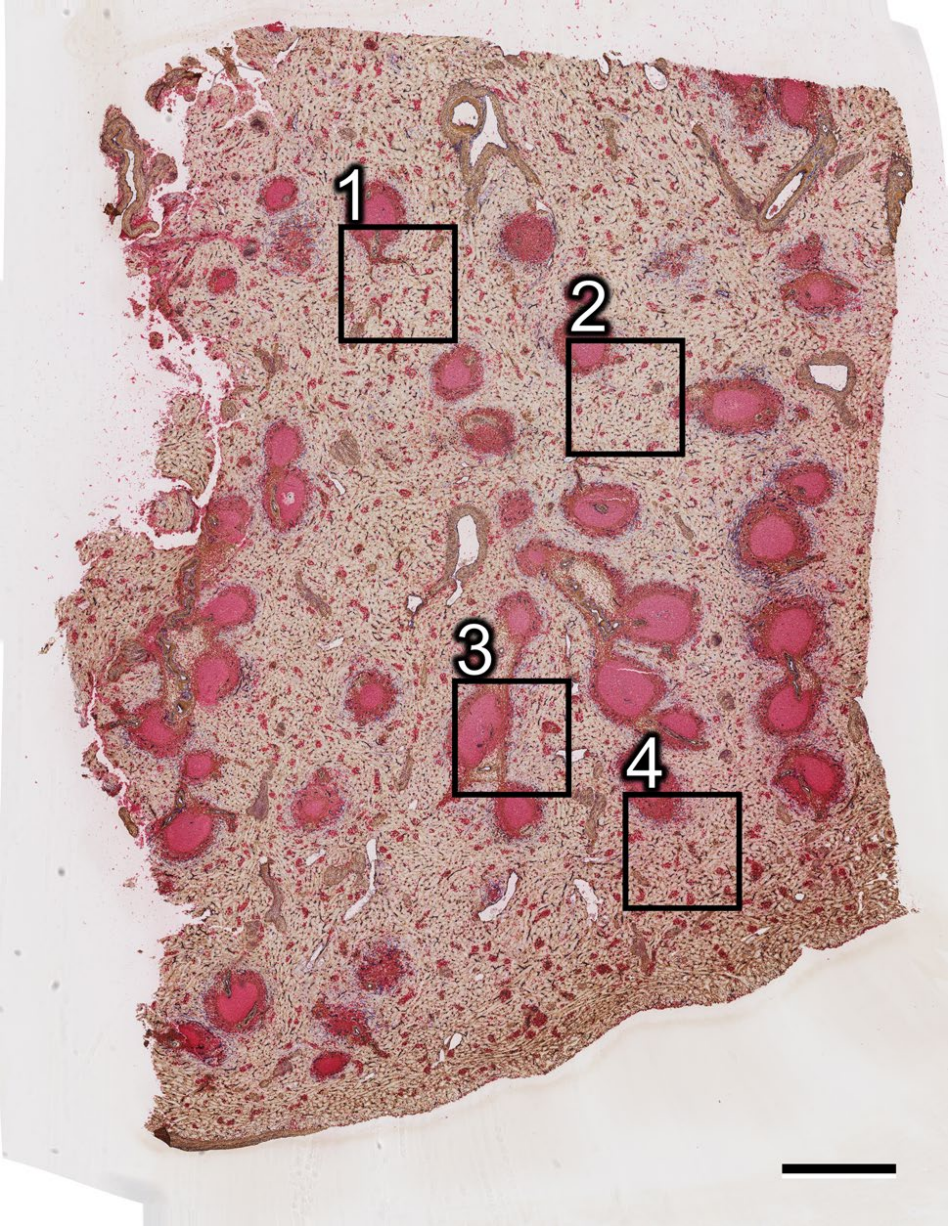
Localization of ROI 1–4 in section 1. Bar = 1 mm

**Fig. 2:**
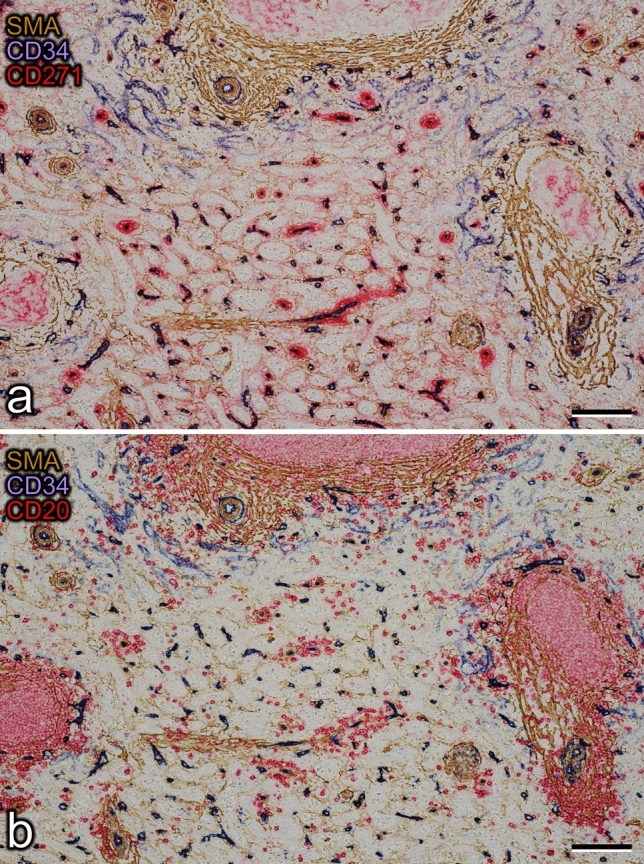
Immunohistological triple staining of serial sections. **a** Demonstration of SMA (brown), CD34 (blue) and CD271 (red) in section 37. Stromal capillary sheath cells and parts of three follicles with CD271^+^ FDCs are visible in red. Note weak expression of CD34 in perifollicular sinuses. The sheaths observed near the surface of the upper follicle and in the lower part of the image are demonstrated in 3D in Fig. 3e, f. **b** Demonstration of SMA (brown), CD34 (blue) and CD20 (red) in section 38 showing B lymphocytes (red) accumulating around the capillary sheaths. The sections were stained in the sequence described. Bars = 100 µm

Four ROIs were arbitrarily chosen in the first serial section (Fig. 1) of a representative adult human spleen and were processed for 3D reconstruction. Three of these ROIs (1, 2 and 4) contained a relatively high amount of red pulp tissue, while ROI 3 included about 50% white pulp. From 83 to 91 sequential sections were used to reconstruct each ROI, which represented a volume from 0.58 to 0.64 mm^3^ (Table 1). Smooth muscle alpha-actin (SMA) was first demonstrated in brown, showing the smooth muscle layer of arteries and arterioles, periarterial branched connective tissue cells and a superficial layer of branched fibroblasts surrounding follicles as well as cells inside trabeculae (Fig. 2a, b). The antibody dilution applied was optimized to avoid ubiquitous interstitial staining of fibroblasts in the red pulp. CD34 was subsequently shown in blue. The mAb detected endothelial cells in all vessels with the exception of most sinuses. Only in the vicinity of follicles were sinus endothelia weakly stained. In addition, CD34 occurred in very few adventitial fibroblasts forming rings around larger arteries. It was also weakly present in cells at the surface of trabeculae (Fig. 2a, b). CD271 was most strongly expressed in stromal capillary sheath cells and in follicular dendritic cells (FDCs), which were stained red in the third step (Fig. 2a). The anti-CD271 antibody dilution chosen minimized weak staining in ubiquitous perivascular adventitial fibroblasts and in interstitial fibroblasts of the red pulp. In every other section, CD20 was used to detect B lymphocytes in red color together with SMA in brown and CD34 in blue to permit aligning the sections (Fig. 2b).

**Table 1:** Number of sheaths and follicles in the four ROIs

	Follicles	All sheaths	Complete sheaths	Volume of ROI (mm^3^)
ROI 1	2	187	107	0.58
ROI 2	2	186	105	0.58
ROI 3	2	229	120	0.64
ROI 4	3	113	51	0.59

### Arrangement of terminal arterioles and capillary sheaths

Our aim was to correctly visualize the arterial side of the human splenic microvasculature, which is inadequately represented in the present Terminologia histologica (Federative International Committee on Anatomical Terminology 2008) and in many histology textbooks. Especially sheathed capillaries are a problem, because they do not form short elliptical structures of uniform cell composition as generally assumed. This assumption is due to the lack of immunohistological information about sheath composition and to missing 3D reconstructions. In addition, we wanted to clarify whether each post-arteriolar capillary carries a sheath and whether there are any morphological indications of sheath function.

**Fig. 3:**
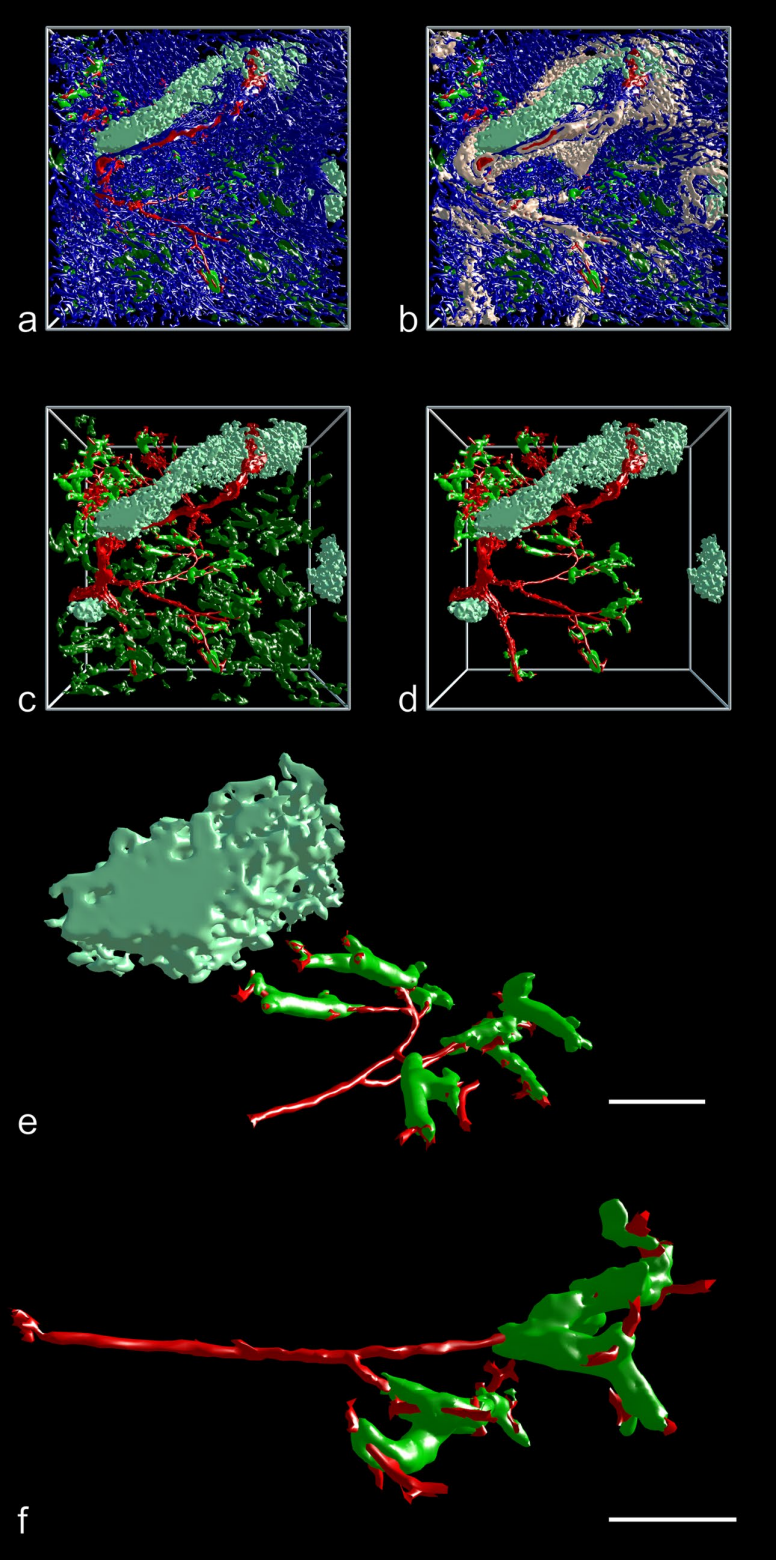
Afferent arterial vessels (red) running to capillary sheaths (green). 3D model with section 84 at the front surface of ROI 4 in different stages of removal of internal structures mimicking VR with front plane clipping. **a** Entire model showing high self-occlusion. The large structures in light green represent CD271^+^ FDC networks in follicles. The follicle in the upper part of the image is exceptional, because it is elongated and not round. **b** Same model with addition of SMA-positive cells in white. The white cells comprise the most strongly stained smooth muscle cells in vessel walls and a large population of perifollicular as well as perivascular fibroblasts. **c** Model without the red pulp capillary network to reduce self-occlusion. **d** Same model without unrelated sheaths. **e** Isolated terminal arteriole with sheaths at the surface of the elongated follicle in the upper part of the model. Capillary sheaths near follicles often bend back into the direction of the feeding arteriole. The post-sheath capillaries feed a perifollicular capillary network not depicted here. **f** Straight terminal arteriole with sheaths from the lower part of the model. Capillary sheaths often occur at terminal branching points of arterioles. They may be V-shaped or may cover each arising capillary separately. Red: selected terminal arteries/arterioles and sheathed capillaries; blue: CD34^+^ endothelia of capillaries, larger vessels and few periarterial fibroblasts; light green: CD271^+^ FDCs; green: CD271^+^ sheaths fed by the selected arterioles; dark green: all other CD271^+^ sheaths in the ROI. Red: manual labeling, green and blue: automatic labeling; dark and light green: semi-automatic labeling. The X- or Y-axes of the bounding boxes represent 1 mm. **e**, **f** Bar = 100 µm

**Fig. 4:**
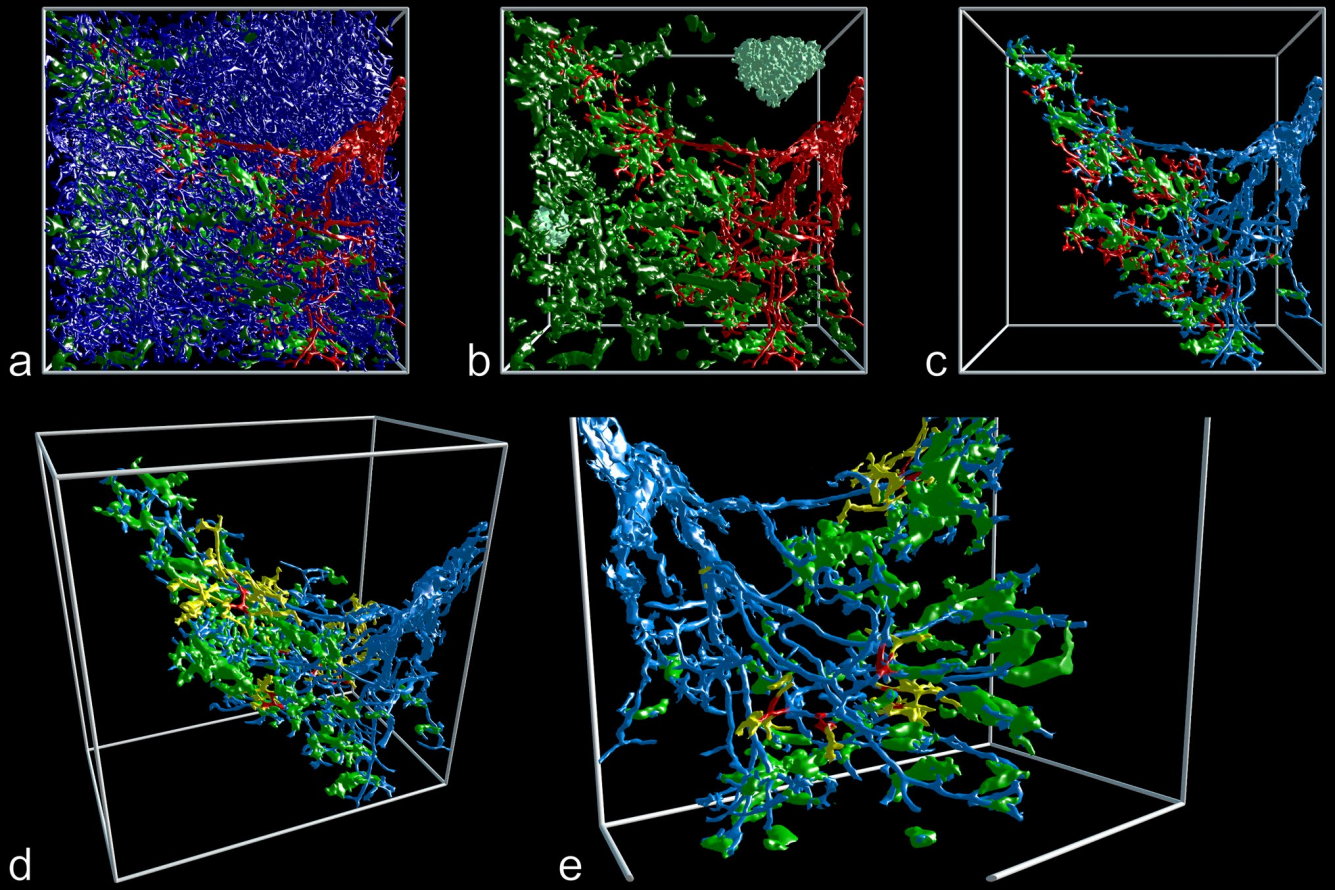
Open-ended side branches of sheathed capillaries (red, **a**–**c**) and non-sheathed capillaries connecting terminal arterioles to the red pulp capillary network (red, **c**–**e**). 3D model with section 83 at the front surface of ROI 2 showing stepwise removal of 3D structures (**a**–**c**). **a** Entire model showing high self-occlusion. **b** Model without red pulp capillary network to reduce self-occlusion. **c** Sheathed capillaries in detail. Red: open-ended side-branches of sheathed capillaries; green: associated sheaths; blue: selected terminal arteries/arterioles. The red side-branches are depicted in their entire length. They appear to irrigate the surfaces of the sheaths. The red pulp capillary network is not included. Sheaths cut at the surface of the ROI are not fully visualized. **d** Non-sheathed connections of terminal arterioles to the red pulp capillary network highlighted in red. Blue: selected terminal arterioles and sheathed capillaries; yellow: parts (200 µm) of the red pulp capillary network directly supplied by a red non-sheathed capillary. **e** Same area as (**d**), but enlarged and observed from the backside of the ROI. **a**, **b** Red: Selected SMA^+^ terminal arteries, their terminal arterioles and sheathed capillaries; blue: CD34^+^ endothelia of red pulp capillary network, larger vessels and few periarterial fibroblasts; green: CD271^+^ capillary sheaths fed by the selected arterioles; dark green: all other CD271^+^ sheaths in the ROI; light green: CD271^+^ FDCs in follicles. Red (**a**–**e**) or blue (**c**–**e**): manual labeling; green and blue (**a**, **b**): automatic labeling; dark and light green: semi-automatic labeling. The X- or Y-axes of the bounding boxes represent 1 mm

We thus decided to track sheathed capillaries arising from terminal arterioles by mesh painting, because this permitted to unequivocally fix the origin of the vessels investigated. The number of capillary sheaths (complete sheaths and sheaths cut at the surface of the ROI) ranged from 113 to 228 per ROI (Table 1). Terminal arterioles were defined as vessels branching from small arteries of larger diameter, containing smooth muscle cells in their walls and terminating in capillary sheaths. Such arterioles were observed to either form a penicillar structure at their origin or to occur as successive single side branches from a terminal artery (Figs. 3a–f, 4a, b). Analyzing the cause of this variability within a ROI was impossible and would have needed reconstruction of the entire section.

Capillary sheaths consisted of a single layer of CD271^+^ stromal sheath cells, which mimicked a cuboidal epithelium (Fig. 2a). These sheath cells were surrounded and infiltrated by B lymphocytes and macrophages, as described previously (Steiniger et al. 2014). The sheaths varied in shape, length and height. The simplest structure of a terminal arteriole was a stem, which either gave off capillary side branches that were each covered by a sheath, or arteriolar side branches, which exhibited sheaths at some distance from the stem. The arteriolar stem often gave off two larger branches, leading to capillary sheaths and it often had a terminal bifurcation or trifurcation after which capillary sheaths started (Fig. 3a-d, Suppl. Videos 1–4, Suppl. Videos 1–3). Sheaths had a tendency to immediately start after a branching point, but sometimes also entirely covered this point (Figs. 3f, Suppl. Videos 2–4, Suppl. Videos 1–3). There were, however, multiple variations with respect to the arrangement of sheaths.

In general, capillary sheaths began where the smooth muscle cells of the arteriole ended. However, pericytes in sheaths also exhibited SMA and, in addition, it could not be totally excluded that the CD271^+^ stromal capillary sheath cells were faintly SMA^+^. Thus, in rare occasions, the most distal part of the feeding arteriole might have also been contained in a sheath.

**Fig. 5:**
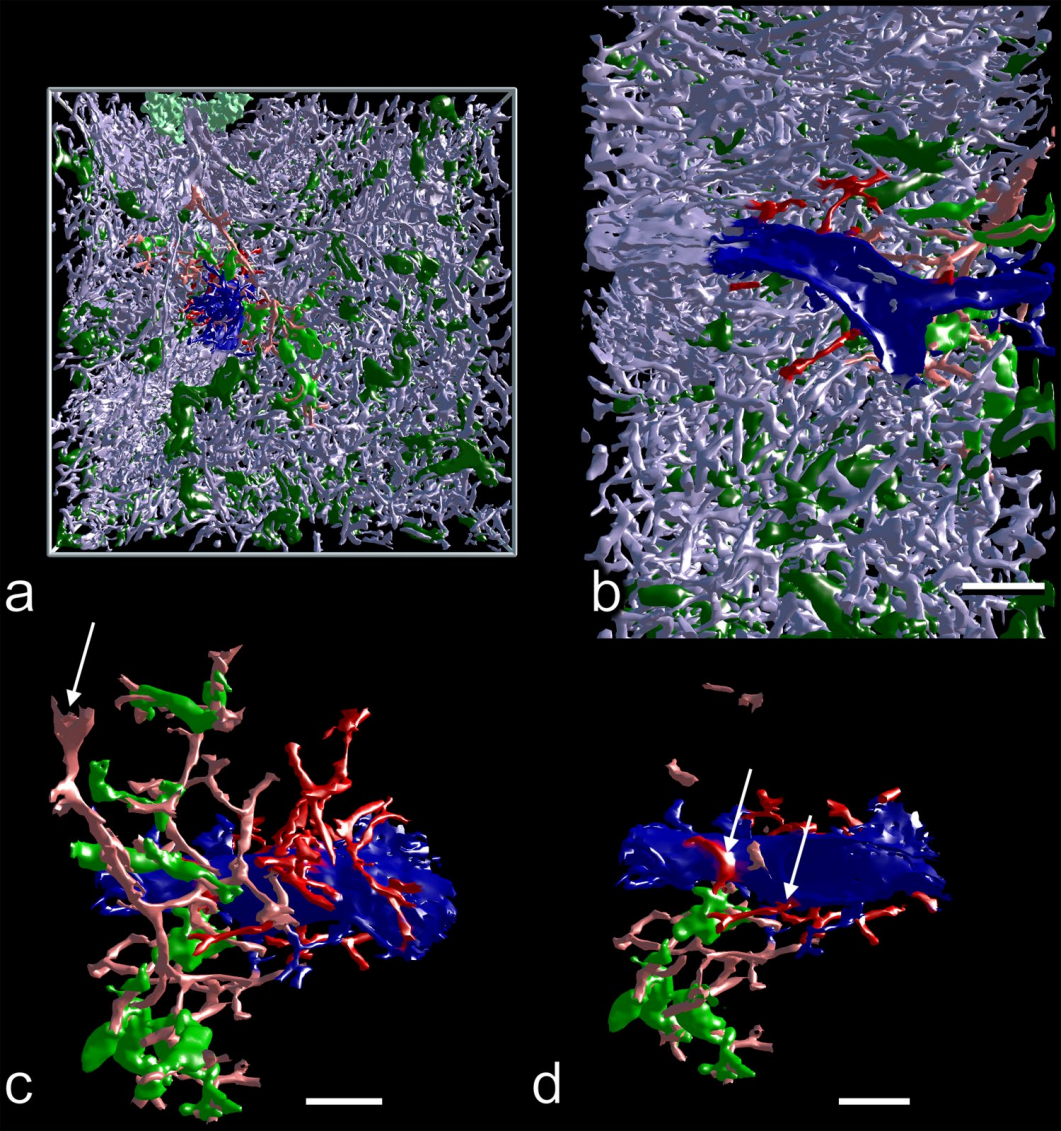
Apparent connections (dark red) of the red pulp capillary network (light blue) to a red pulp venule (dark blue). 3D model with section 1 at the front surface of ROI 1 showing a terminal arteriole (light red) with associated capillary sheaths (green). **a** Overview of ROI 1. Dark green: unrelated sheaths. Blue: CD34^+^ red pulp venule and capillaries extremely close to this vessel, but not unequivocally connected. Dark red and dark blue capillaries are only labeled close to the venule. The X- or Y-axis of the bounding box represents 1 mm. **b** ROI 1 with same red pulp venule observed in the YZ plane. The venule (dark blue) has been sectioned longitudinally. The venule and close capillaries of uncertain relation to this vessel are only labeled in the right part of the image. The venule is fed by sinuses and initially lacks CD34^+^ endothelial cells (right side of image). **c** Higher magnification of (**a**, **b**) without major parts of the capillary network and with selection of a part of the venule (section 1–60) seen from outside. All capillaries were cropped arbitrarily at some distance from the venule. Post-sheath capillaries leading into the red pulp capillary network were cropped arbitrarily at some distance from the sheath. Labeling as indicated for (**a**, **b**). Arrow shows terminal artery/arteriole (light red) feeding the sheaths depicted. **d** Same image as (**c**), but with feeding arteriole and further parts of capillaries removed to avoid superpositioning. Arrows show two capillaries leaving a sheath, which are apparently connected to the venule. Red colors: manual labeling; light blue, blue and green: automatic labeling; dark and light green: semi-automatic labeling. Scale bars in (**b**–**d**) = 100 µm

Large sheaths covered up to four sequential dichotomous branchings of the post-arteriolar capillary before the capillary finally joined the red pulp capillary network or had open ends. The shape of sheaths was bizarre and sometimes also involved a U-shaped turn, so that a sheathed capillary ran back toward its feeding arteriole. This was often seen at the surface of the white pulp, where capillaries leaving their sheaths joined the superficial capillary net of the follicles or T cell zones (Fig. 3c–e, Suppl. File 2, Suppl. Videos 2, 3). In most cases, only one central capillary was contained in a sheath, but on closer inspection, the capillaries were observed to release asymmetric side branches inside their sheaths (Suppl. File 6). These side branches often directly crossed the stromal sheath cells and ended at their surface (red in Fig. 4c). Some also ran parallel to the main capillary inside the sheath, but finally passed through the sheath and ended. This indicates that the side branches had open ends. Longer side branches even formed ring-shaped or longitudinal vessels running and ending at the sheath surface without joining the extra-sheath capillary network. Anastomoses to the network also occurred, but they were rare. The vast majority of capillary branches leaving a sheath (red in Fig. 4c) terminated at the surface of the sheath or at some distance from the sheath. In rare cases, anastomoses formed an indirect connection to another sheathed capillary via the network or even a connection to a venule (white arrows in Fig. 5d, Suppl. File 10).

Terminal arterioles also exhibited side branches joining the capillary network of the red pulp without intercalated sheaths (red parts of vessels in Fig. 4d, e, Suppl. File 7). Such non-sheathed connections were always present, but sheathed capillaries were much more numerous.

### Association of arteries, arterioles and capillary sheaths with B lymphocytes

**Fig. 6:**
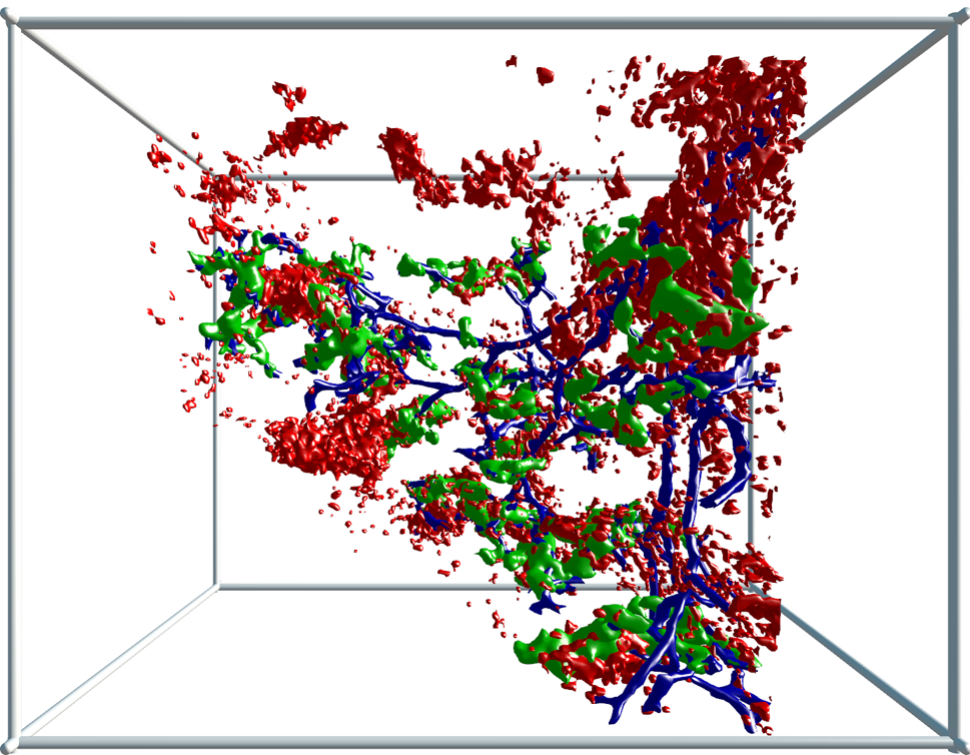
Accumulations of CD20^+^ B lymphocytes (red) around a selected terminal artery (blue) and around capillary sheaths (green). 3D model of the lower half of ROI 2 with section 83 at the front surface. The isolated B cell accumulations in the upper left part of the image are associated with capillary sheaths fed by terminal arterioles not arising from the labeled terminal artery. Blue: manual labeling; green and red: automatic labeling. Irrelevant B lymphocytes were removed for improved visualization (see “Materials and methods”). Dimensions of bounding box: 800 (b) × 620 (h) × 614 (d) µm

When the volume filled by CD20^+^ B lymphocytes (red in Fig. 6) was included in the 3D model, it became clear that B lymphocytes did not only cover the CD271^+^ stromal sheath cells, but they also infiltrated these cells and were present in direct contact to the endothelium (Fig. 2b, Suppl. Files 8, 9, Suppl. Video 4). Thus, B lymphocytes formed a regular constituent of capillary sheaths. As published previously (Steiniger et al. 2014), the phenotype of the B cells indicated that they represented small recirculating naive cells. In general, larger sheaths with large stromal sheath cells tended to be associated with more B lymphocytes than smaller sheaths with small stromal sheath cells, which suggests a close interdependence of both cell types. Larger sheaths appeared to be associated with the terminal branches of arterioles, while smaller sheaths tended to cover side branches.

When only B lymphocytes (red in Fig. 6) and arterial vessels (blue in Fig. 6) were visualized in the models and compared to the original sections (Fig. 2b), it was evident that there were two major regions, where B lymphocytes accumulated in the red pulp, namely around small arteries/non-terminal arterioles and around capillary sheaths. The arteries/arterioles were those which gave rise to terminal arterioles. Thus, the starting points of these arterioles were sometimes included in the periarterial B cell cuffs (accumulation of red cells in the right upper part of Fig. 6, Suppl. Files 8, 9, Suppl. Video 4). Interestingly, with the exception of these starting points, the terminal arterioles were not accompanied by major numbers of B cells until the sheaths started. Our present and previous (Steiniger et al. 2014) observations show that the small pre-arteriolar vessels are surrounded by a very characteristic network of SMA^+^ fibroblasts (lower left and right parts of Fig. 2a, b). This network does not only contain B lymphocytes, but a mixture of B and T cells (Steiniger et al. 2014).

### Sheath-associated macrophages

**Fig. 7:**
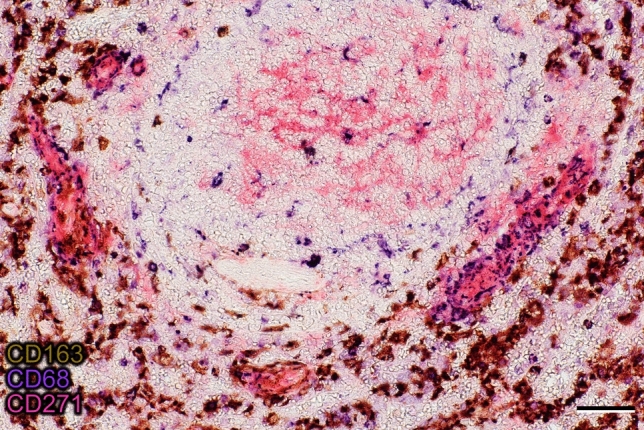
Phenotype of macrophages in a follicle and in perifollicular capillary sheaths. In contrast to CD163^+^ sheath-associated and non-associated macrophages in the red pulp (brown), macrophages in a follicle (center of image) and in three perifollicular capillary sheaths are predominantly—but not exclusively—CD163^−^CD68^+^ (blue). CD271^+^ stromal sheath cells and FDCs in the follicle are stained red. The unstained area in the lower part of the follicle is a small artery. The section was stained in the sequence CD163 (brown), CD68 (blue), CD271 (red) as indicated. Bar = 50 µm

Sheath-associated macrophages were not included in the models of the present investigation, because their phenotype is heterogeneous and some of them may be difficult to distinguish from red pulp macrophages. We did, however, try a preliminary phenotypic analysis of sheath-associated macrophages in different double-staining experiments, showing that sheaths close to white pulp areas must be distinguished from sheaths deep in the red pulp. Present and previous (Steiniger et al. 1997) results indicate that the predominant macrophage phenotype in perifollicular sheaths is CD68^+^CD169^+^ CD163^−^, while red pulp sheaths mainly contain CD68^+^CD169^±^CD163^+^ macrophages (Fig. 7). In human spleens, the CD68^+^CD163^−^ phenotype is typically associated with the white pulp; most red pulp macrophages are CD68^+^CD163^+^. The macrophages do not only surround the CD271^+^ stromal sheath cells, but they also infiltrate between them. In fact, a dense granular staining pattern for CD68 (Fig. 7) or for CD163 can be found within the area stained for CD271. These granules appear more densely than in the surrounding red pulp and may indicate that sheath-associated macrophages are more dendritic than ordinary red pulp macrophages.

### Red pulp venules

Red pulp venules were easily detectable, because they had a diameter larger than sinuses and were predominantly lined by a CD34^+^ endothelium, while most red pulp sinuses were CD34^−^. However, the initial parts of venules had patches of CD34^−^ endothelium (right part of blue-stained vessel in Fig. 5b), which were either CD141^+^ or exhibited a mixed phenotype (Steiniger et al. 2007). Sinuses were often observed opening into such initial venules. The 3D models seemed to show that the venules were connected to the red pulp capillary network, but such connections were rare (Fig. 5b). Capillaries often ran parallel to the venules and finally ended extremely close to the venular endothelium, so that a connection was highly likely, but difficult to recognize in detail (dark red vessels in Fig. 5a–d, Suppl. File 10, Suppl. Video 5). Thus, there were a number of capillaries the ends of which could not be unequivocally identified in relation to the nearby venule. It could not be excluded that the ends of collapsed capillaries had artificially dropped onto the venules when perfusion pressure was no longer present. In rare instances, a capillary leaving a sheath appeared to directly join a venule (Fig. 5c, white arrows in Fig. 5d, Suppl. File 10, Suppl. Video 5). In any case, the low number of connections indicated that most vessels of the red pulp capillary network did not deliver their blood to venules. Thus, the microcirculation of the human splenic red pulp has potential closed parts, but the open circulation predominates.

## Discussion

Our 3D reconstructions demonstrate that sheathed capillaries are specialized vascular structures located distal to branching points of terminal arterioles in the human splenic red pulp. The sheaths consist of capillary endothelial cells and pericytes surrounded by special CD271^+^ stromal sheath cells mimicking a cuboidal epithelium, by B lymphocytes and by macrophages. Single sheaths cover up to four sequential bifurcations of a post-arteriolar capillary before this vessel joins the splenic cord capillary network or has open ends. The shape of capillary sheaths is elongated and not ellipsoidal. Some sheaths form bizarre branching structures covering a central vessel for a distance of more than 300 µm, measured by geodesic distance from its entry to its exit. Direct non-sheathed connections of arterioles to the red pulp capillary network also occur, but sheathed capillaries prevail.

Our results unequivocally show that the sheathed vessels need to be termed "sheathed capillaries" and not "sheathed arteries" or "sheathed arterioles". Sheath formation only starts after birth (own unpublished observation). Thus, the presence of post-arteriolar capillary endothelium and of exogenous antigens is required for sheath formation.

Sheathed capillaries are surrounded by open blood-filled spaces of splenic cords and by accumulations of B lymphocytes and macrophages. They directly deliver blood to these spaces and to the surrounding cells via short open-ended asymmetric side branches. Most terminal capillary branches leaving sheaths are, however, also of limited length and connections to the red pulp capillary network are rare. In the splenic circulation, sheathed capillaries represent the first structures where the endothelium is confronted with soluble materials from the incoming blood both from its apical and basal side. This fact may provoke the existence of sheaths at locations where capillaries branch in very short succession. Capillary branching decelerates the blood flow and provokes turbulences which guarantee an even distribution of different blood components to the open side branches of the sheathed capillaries. Thus, we hypothesize that sheathed capillaries are hubs for distributing incoming antigens, small particles, etc., to their surface. The immunocompetent cells predisposed to deal with soluble antigens are B lymphocytes and macrophages, but not T lymphocytes. In consequence, B cells, but not T cells, are associated with capillary sheaths.

Splenic capillary sheaths have been neglected in research, because such structures are absent in mice and rats. This is, however, an exception in evolution, because most other species possess sheaths. In fishes and birds, the equivalents of capillary sheaths, the so-termed ellipsoids, are locations where particulate foreign materials, antigens, immune complexes and pathogens are immobilized very soon after intravenous injection (Eikelenboom et al. 1983; Espenes et al. 1995; Jeurissen 1993; Lamers and de Haass 1985; Soerby et al. 2005). Ellipsoids have been more precisely explored in birds, where they give rise to a second normal splenic B cell compartment in addition to follicles (Olah et al. 1984, 2013; Kasai et al. 1995; Mast and Goddeeris 1998), termed the periellipsoid lymphatic sheaths (PELSs). Human capillary sheaths appear to represent miniaturized versions of PELSs found in chickens. The arrangement and form of human and avian stromal sheath cells are very similar (Mast and Goddeeris 1998), but the number of B lymphocytes is much less in humans.

We looked for additional molecules besides CD271, which might be predominantly expressed in stromal sheath cells, but the results were disappointing. In cryosections, CD90 (Thy-1) appeared to be present; however, CD90 has a lipid anchor and thus needs to be visualized in properly fixed material. Up to now, we have not found any anti-CD90 reagent for paraffin sections. Additional molecules typical of FDCs, such as CD21, CD35, or the unknown target of mAb CNA.42 (Raymond et al. 1997), were not detected in sheaths. This was also true for two pathological spleens of patients with immune thrombocytopenia.

**Fig. 8:**
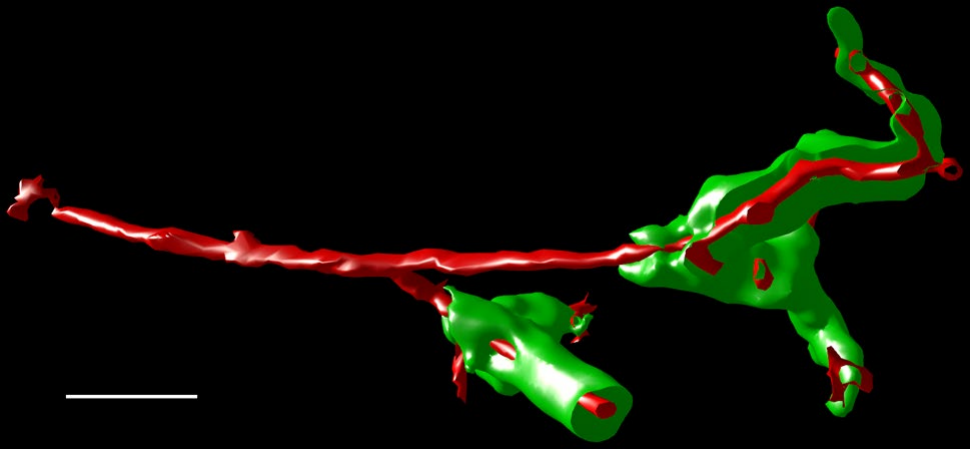
Isolated feeding terminal arteriole and capillary sheaths opened by front plane clipping. Same arteriole as shown in Fig. 3f. Bar = 50 µm

The most convenient way of inspecting the models is by VR, because the observer is totally immersed into the model and can continuously and intuitively move through a vessel or a sheath simultaneously viewing it both from outside and from inside with the help of front plane clipping (Fig. 8). Our application can also be used for interactive desktop inspection of the models in a 2D or 3D screen, but this needs constant manipulations. Searching a proper camera position with front plane clipping is very exhausting in this case. If VR is used, viewpoint adjustments are, however, trivial. This is especially relevant, because the original registered sections have to be constantly switched on and off for quality control during inspection. We thus strongly recommend using VR to avoid fatigue. Referring to the starting material, i.e., being able to blend each immunostained section into the model, is absolutely mandatory to avoid interpretation errors when analyzing complicated vascular networks. Combining internal and external aspects of sophisticated structures such as sheaths is also decisive. Our reconstruction method for multiple colors is optimal for small vessels, because they are compact and only the outer surface is rendered. It may, however, lead to complications in larger vessels such as arteries. In this case, the lumen is an entity separate from the blood vessel wall, which leads to both the outer and inner surface of the stained area being visualized for each color. Thus, the marching cubes algorithm produces a multi-layered envelope around the vessel, which is difficult to interpret. In addition, processing of the mesh data may lead to spaces closed across the lumen of large vessels which appears as an interruption of their internal structure.

By mesh painting, display of the original stained sections within the 3D model, and by the front plane clipping adjustment for highly occluded regions, our VR application (Steiniger et al. 2018; Berthold 2017; Lobachev 2018) represents a novel combination of methods, which extends the range of similar tools independently introduced by others (Egger et al. 2017; El Beheiry et al. 2019).

We used immunostaining for transmitted light, because this gives optimal morphological information and provides specimens which are permanent. In our hands, this method did, however, limit the number of colors in a single section to three. To visualize a fourth antigen, we varied the antigen depicted in red color in every other section while retaining the other two colors and then interpolated the red channel. This was obvious, because interpolation was a typical part of the processing pipeline used to improve the resolution in direction of the z axis.

We did not find any indication that the cutting process provoked a loss of material between two consecutive sections, as far as registration and 3D reconstruction were concerned. Even a delicate structure such as an extension of an isolated endothelial cell at a capillary end could be visualized.

Our approach might be improved in several ways. Partially or totally out-of-focus scans may occur with the Leica SCN 400 scanner and interrupt reconstruction of vessels, if a section contains uneven structures. This problem is likely to be reduced in more advanced scanning systems. Automatized vessel repair is now possible (Lobachev 2020). Further improvements, e.g., augmenting the method with deep learning or subsequent VR-based quality control, would allow for an even wider utilization. Machine learning and advanced threshold-based methods might also be viable to separate CD34^++^ capillaries and weakly CD34^+^ perifollicular sinuses. Threshold-based methods do, however, require a lot of experiments, and machine learning needs a substantial quantity of annotated data, even when using generative adversarial networks. In the absence of undesired sinus labeling, more automatic and semi-automatic markup approaches, e.g., based on geodesic distances, can be used. Avoiding manual mesh painting to track arteries, arterioles or other structures would save a lot of time and abolish errors. A tool that allows for tracing larger structures over hundreds and thousands of micrometers would be an important software component not only in the context of splenic microanatomy, but also for the study of other organs. In addition, early in the pipeline, deep learning-based methods on the normalization of serial sections might permit producing even better inputs.

Currently, the amount of data for all four ROIs amount to hundreds of gigabytes. This is in the range of commodity hardware. Processing of full sections would need multiple terabytes of hard disk space and corresponding processing times. With respect to interactive visualization in VR, the limitation is the available memory of graphics accelerators. Presently, this problem can only be overcome with considerable programming efforts using new and existing methods in texture compression and progressive meshes. For example, the immediate results of marching cubes in this work would occupy less space if improved methods on parallel construction and simplification were used, similar to those designed by Attali et al. (2005) or Ulrich et al. (2014).

The results reported in this study are based on surface mesh representations. This is the most widely used representation in computer graphics based on commercially available rasterization accelerators which are used in gaming. Thus, surface representations are most straightforward to present in virtual reality. Instead, we might use raw volume-based representations without application of marching cubes or construct volume meshes. These methods would potentially solve the problems with double contours in large blood vessels. The disadvantage of such an approach is that we would not be able to use standard rasterization-based renderers and would eventually not be able to use commodity hardware.

Up to now, only single experts can be immersed in VR and all other participants of a session need to watch the expert´s field of view by means of an external display, which yields limited information. A further goal might be to adapt the software to multi-user VR sessions so that experts may cooperate and discuss the models while being immersed.

In spite of benefiting from future improvements, our investigation yielded unique insights into special parts of the splenic microvasculature. One of the most interesting findings are the side branches originating from the capillaries inside or after the sheaths. Most of the side branches tend to be short and to supply the surface of the sheath via open ends. However, long recurrent straight or ring-shaped superficial branches of sheathed capillaries do also occur. Thus, macrophages and B lymphocytes are immediately confronted with blood arriving via terminal arterioles. As mentioned above, a part, but not all, of the sheath-associated macrophages exhibits a special phenotype typically associated with splenic white pulp macrophages, namely CD68^+^CD163^−^. A more detailed phenotypic analysis of sheath-associated macrophages deep in the red pulp might unravel whether and how these cells differ from the surrounding ubiquitous macrophages in the splenic cords. Up to now, we have not been able to detect any materials stored or bound by these macrophages, but it is very likely that such substances can be found. It may be that a larger number of pathologic spleens needs to be investigated to detect antigens or immune complexes in sheath-associated macrophages.

The sheath-associated B lymphocytes exhibit the typical phenotype of naive recirculating cells. They express CD20, represent switched and non-switched cells and are negative for CD27. Thus, capillary sheaths seem to be involved in the recirculation pathway of B lymphocytes through the spleen. It may very well be that not only materials delivered to the spleen by the arterial blood, but also recirculating B lymphocytes are retained for some time in the sheaths, before they approach the superficial T cell zone and then enter the mantle zone of follicles. Some time ago we demonstrated that a potent B cell-attracting chemokine, CXCL13, is abundantly present in stromal capillary sheath cells (Steiniger et al. 2014). It could be envisaged that B cells exit the blood stream through side branches of sheathed capillaries, stop in association with the sheaths and then migrate in a retrograde fashion to reach the B cell clusters around small arteries/non-terminal arterioles. However, our models show only very few B lymphocytes around terminal arterioles. Thus, retrograde migration along these arterioles could be very fast, because—in contrast to sheaths or small arteries/arterioles—no other immunocompetent cells are present.

Our results show that the human splenic microcirculation is both open and closed, but that the open part is predominant. We derive this opinion from the observation of only few connections between the red pulp capillary network and red pulp venules. We suppose that these connections form a kind of "emergency exit" in case the drainage of blood from the open spaces of the splenic cords into the sinuses is obstructed. It can, however, not be totally excluded that capillaries which are found close to venules do not join these vessels at all, but just end extremely close to their surface. This problem arises, because the wall of smaller venules is very thin and only consists of endothelial cells. Our study is not aimed at studying potential connections between capillaries and sinuses, because we did not use antibodies detecting sinus endothelia. Up to now, we have not observed capillaries joining sinuses by more conventional methods. However, this question needs to be resumed by constructing 3D models from sections double stained for CD34 and for CD141 or other sinus-associated antigens as published previously (Steiniger et al. 2011), but applying VR for their evaluation. VR is essential for investigating and labeling long structures inside space-filling models. A prospective reconstruction of sinuses cannot be properly evaluated without VR.

Stromal sheath cells may serve to concentrate macrophages and B lymphocytes to the place where blood-borne antigens first enter the spleen. The B-lymphocyte-attracting chemokine CXCL13 is likely to be one of the mediators involved (Steiniger et al. 2014), but it has to be expected that there are many more. Interactions with macrophages may provide some form of non-specific signal to B lymphocytes, which pick up antigen in the sheaths. This may promote antigen-specific interaction with T helper lymphocytes in the mixed clusters surrounding larger arteries/arterioles, if the antigen-bearing B lymphocytes have reached this site. The variable size and number of stromal capillary sheath cells indicate that the interaction of the three sheath-associated cell types is highly dynamic.

## Data Availability

The data are available from https://zenodo.org/record/4059595.

## References

[CR1] Ahrens J, Geveci B, Law C, Hansen CD, Johnson CD, ParaView (2005). An end-user tool for large data visualization. Visualization handbook.

[CR2] Alt H, Guibas LJ (1999) Discrete geometric shapes: Matching, interpolation, and approximation. In: Sack J-R, Urrutia J (eds) Handbook of computational geometry, pp 121–153

[CR3] Attali D, Cohen-Steiner D, Edelsbrunner H (2005) Extraction and simplification of iso-surfaces in tandem. In: Fellner D, Spencer S (eds) Proc. SGP ’05, Eurographics*,* pp 139–148.

[CR4] Ayachit U (2015). The ParaView guide: A parallel visualization application.

[CR5] Berthold M (2017) VR-based visualization of medical data. BSc Thesis (University of Bayreuth)

[CR6] Cignoni P et al. (2008) Meshlab: An open-source mesh processing tool. In: Scarano V, De Chiara R, Erra U (eds) Eurographics Italian chapter conference, pp 129–136

[CR7] Drenckhahn D, Wagner J (1986). Stress fibres in the splenic sinus endothelium in situ: molecular structure, relationship to the extracellular matrix, and contractility. J Cell Biol.

[CR8] Egger J (2017). HTC Vive MeVisLab integration via OpenVR for medical applications. PLoS ONE.

[CR9] Eikelenboom P, Kroese FG, van Rooijen N (1983). Immune complex-trapping cells in the spleen of the chicken. Enzyme histochemical and ultrastructural aspects. Cell Tissue Res.

[CR10] El Beheiry M, Doutreligne S, Caporal C, Ostertag C, Dahan M, Masson J-B (2019). Virtual reality: beyond visualization. J Mol Biol.

[CR11] Espenes A, Press CM, Dannevig BH, Landsverk T (1995). Immune-complex trapping in the splenic ellipsoids of rainbow trout (*Oncorhynchus mykiss*). Cell Tissue Res.

[CR12] Farnebäck G (2003) Two-frame motion estimation based on polynomial expansion. In: Bigun J, Gustavsson T (eds) Image analysis, pp 363–370. 10.1007/3-540-45103-X_50

[CR13] Federative International Committee on Anatomical Terminology (2008). Terminologia histologica: international terms for human cytology and histology.

[CR14] Jeurissen SH (1993). The role of various compartments in the chicken spleen during an antigen-specific humoral response. Immunology.

[CR15] Ju T (2004). Robust repair of polygonal models. ACM T Graphic.

[CR16] Kasai K, Nakayama A, Ohbayashi M, Nakagawa A, Ito M, Saga S, Asai J (1995). Immunohistochemical characteristics of chicken ellipsoids using newly established monoclonal antibodies. Cell Tissue Res.

[CR17] Khan AM, Rajpoot N, Treanor D, Magee D (2014). A nonlinear mapping approach to stain normalization in digital histopathology images using image-specific color deconvolution. IEEE T Biomed Eng.

[CR18] Kikinis R, Pieper SD, Vosburgh KG, Jolesz FA (2014). 3D Slicer: A platform for subject-specific image analysis, visualization, and clinical support. Intraoperative imaging and image-guided therapy.

[CR19] Kotzé HF, Heyns AD, Wessels P, Pieters H, Badenhorst PN, Lötters MG (1986). Evidence that ^111^In-labelled platelets pool in the spleen, but not in the liver of normal humans and baboons. Scand J Immunol.

[CR20] Lamers CH, De Haas MJ (1985). Antigen localization in the lymphoid organs of carp (*Cyprinus carpio*). Cell Tissue Res.

[CR21] Lobachev O (2018) On three-dimensional reconstruction. Habilitationsschrift (University of Bayreuth), https://epub.uni-bayreuth.de/3774

[CR22] Lobachev O (2020). The tempest in a cubic millimeter: Image-based refinements necessitate the reconstruction of 3D microvasculature from a large series of damaged alternately-stained histological sections”. IEEE Access.

[CR24] Lobachev O, Steiniger BS, Guthe M (2017a) Compensating anisotropy in histological serial sections with optical flow-based interpolation. In: Spencer, SN (ed) Spring conference on computer graphics, 14:1–14:11, ACM

[CR23] Lobachev O, Ulrich C, Steiniger BS, Wilhelmi V, Stachniss V, Guthe M (2017). Feature-based multi-resolution registration of immunostained serial sections. Med Image Anal.

[CR25] Lorensen WE, Cline HE (1987). Marching cubes: A high resolution 3D surface construction algorithm. SIGGRAPH Comput Graph.

[CR26] Mast J, Goddeeris BM (1998). CD57, a marker for B-cell activation and splenic ellipsoid-associated reticular cells of the chicken. Cell Tissue Res.

[CR27] Olah I, Glick B, Taylor RL (1984). Effect of soluble antigen on the ellipsoid-associated cells of the chicken´s spleen. J Leukoc Biol.

[CR28] Olah I, Nagy N, Vervelde L, Schat K, Kaspers B, Kaiser P (2013). Structure of the avian immune system. Avian Immunology.

[CR29] Onder D, Zengin S, Sarioglu S (2014). A review on color normalization and color deconvolution methods in histopathology. Appl Immunohistol Mol Med.

[CR30] Pieper S, Halle M, Kikinis R (2004) 3D slicer. In: 2nd IEEE international symposium on biomedical imaging: Nano to macro, pp 632–635, IEEE. 10.1109/ISBI.2004.1398617

[CR31] Raymond I, Al Saati T, Tkaczuk J, Chittal S, Delsol G (1997). CNA.42, a new monoclonal antibody directed against a fixative-resistant antigen of follicular dendritic cells. Am J Pathol.

[CR32] Reinhard E, Adhikhmin M, Gooch B, Shirley P (2001). Color transfer between images. IEEE Comput Graph.

[CR33] Ruifrok AC, Johnston DA (2001). Quantification of histochemical staining by color deconvolution. Anal Quant Cytol.

[CR34] Schindelin J, Argnada-Carreras I, Frise E (2012). Fiji: an open-source platform for biological image analysis. Nat Methods.

[CR35] Soerby R, Wien TN, Husby G, Espenes A, Landsverk T (2005). Filter function and immune complex trapping in splenic ellipsoids. J Comp Path.

[CR36] Steiniger BS (2015). Human spleen microanatomy: why mice do not suffice. Immunology.

[CR37] Steiniger B, Barth P (2000). Microanatomy and function of the spleen. Adv Anat Embryol.

[CR38] Steiniger B, Barth P, Herbst B, Hartnell A, Crocker PR (1997). The species-specific structure of microanatomical compartments in the human spleen: Strongly sialoadhesin-positive macrophages occur in the perifollicular zone, but not in the marginal zone. Immunology.

[CR39] Steiniger B, Stachniss V, Schwarzbach H, Barth PJ (2007). Phenotypic differences between red pulp capillary and sinusoidal endothelia help localizing the open splenic circulation in humans. Histochem Cell Biol.

[CR40] Steiniger B, Bette M, Schwarzbach H (2011). The open microcirculation in human spleens: a three-dimensional approach. J Histochem Cytochem.

[CR41] Steiniger BS, Seiler A, Lampp K, Wilhelmi V, Stachniss V (2014). B lymphocyte compartments in the human splenic red pulp: capillary sheaths and periarteriolar regions. Histochem Cell Biol.

[CR042] Steiniger BS, Stachniss V, Wilhelmi V, Seiler A, Lampp K, Neff A, Guthe M, Lobachev O (2016) Three-dimensional arrangement of human bone marrow microvessels revealed by immunohistology in undecalcified sections. PLoS One 11:e0168173. 10.1371/journal.pone.0168173 10.1371/journal.pone.0168173 PMC5172587 27997569

[CR42] Steiniger BS, Wilhelmi V, Berthold M, Guthe M, Lobachev O (2018). Locating human splenic capillary sheaths in virtual reality. Sci Rep.

[CR43] Still M (2006). The definitive guide to ImageMagick. Apress.

[CR44] Taubin G (1995) A signal processing approach to fair surface design. In: Mair SG, Cook R (eds), Proc SIGGRAPH '95, ACM, pp 351–358

[CR45] Ulrich C, Grund N, Derzapf E, Lobachev O, Guthe M (2014) Parallel iso-surface extraction and simplification. In: Skala V (ed), Proc. WSCG, pp 361–368

